# The Impact of COVID-19 on the Orthopaedic Surgery Residency Experience

**DOI:** 10.51894/001c.25963

**Published:** 2021-08-30

**Authors:** Devan O. Higginbotham, Abdul K. Zalikha, Steven K. Stoker, Bryan E. Little

**Affiliations:** 1 Department of Orthopaedic Surgery Detroit Medical Center, Detroit, MI; 2 Department of Orthopaedic Surgery McLaren Oakland, Pontiac, MI

**Keywords:** graduate medical education, orthopaedic residency, united states, pandemic, residency, orthopaedic education, covid-19

## Abstract

**INTRODUCTION:**

The rapid spread of the COVID-19 virus led to dramatic changes in graduate medical education and surgical practice. The purpose of this study was to evaluate the effects of the COVID-19 pandemic on Orthopaedic Surgery residency education in the United States.

**METHODS:**

A survey sent to all residents of the 201 ACGME-accredited Orthopaedic Surgery programs in the United States.

**RESULTS:**

A total of 309 Orthopaedic surgery residents responded to our survey. A subset of 283 (91.6%) residents surveyed reported decreased Orthopaedic-related clinical duty hours due to the COVID-19 pandemic, and 300/309 (97.1%) reported a decrease in surgical case volume. 298 (96.4%) residents reported that their program had scheduled activities or made changes to supplement their education, most common being virtual and video conferences 296/309 (95.5%), required practice questions 132/309 (42.7%), required reading or pre-recorded lectures 122/309 (39.5%), in-person small group meetings or lectures 24/309 (7.77%), and surgical simulation activities 17/309 (5.50%). Almost half (152/309 (48.9%)) of respondents reported their overall resident education was somewhat or much worse due to the impact of COVID-19. Over a quarter (81 (26.2%)) of residents reported their well-being was negatively impacted by residency-related changes due to COVID-19.

**CONCLUSIONS:**

Based on these results, the COVID-19 pandemic has brought about significant changes to the training experience of Orthopaedic surgery residents in the United States. Although the majority of residents in this sample had favorable opinions of the educational changes their programs have instituted in light of the pandemic, clinical duty hours and case volume were reported to have substantially decreased, with a large portion of residents viewing their overall resident education as worsened and reporting negative impacts on their overall well-being.

## INTRODUCTION

The first confirmed case of the novel coronavirus SARS-CoV-2 in the United States was reported on January 20, 2020.^1^ Rapid spread of the virus led to dramatic change in medical and surgical practice. In anticipation of a surge of infected patients as well as increased demand for personal protective equipment (PPE), on March 13, 2020, the American College of Surgeons (ACS) recommended postponing or cancelling elective procedures.^2^ Following these guidelines as well as those of the Centers for Medicare and Medicaid Services, the American Academy of Orthopaedic Surgeons (AAOS) also recommended delaying elective services.^3^

As of July 21, 2021, there were over 35 million confirmed cases of COVID-19 in the United States with total deaths over 625,801.^4^ Due to the pandemic, all surgical specialties were forced to triage the urgency of their surgical caseload and pursue non-surgical options when possible.^5^ Additionally, graduate medical education (GME) normal resident clinical duties and obligations were cut down as a preemptive measure to limit exposure to the virus of essential personnel.

With the decline in clinical and surgical volume due to stay-at-home orders and the postponement of non-emergent surgical cases, the surgical experience of Orthopaedic surgery (OS) residents and fellows has been dramatically affected.^6^ In addition to anticipated changes in clinical and surgical practice, normal educational opportunities such as grand rounds, simulation labs and/or journal clubs underwent changes or elimination as program directors implemented changes to meet national guidelines and governmental orders in efforts to preserve the health of residents. To date, little is known regarding the lasting effects of the COVID-19 pandemic on the GME experiences, well-being, and competency of OS residents.

### Purpose of Study

The purpose of this 2020 cross-sectional survey project was to examine the perceptions of current OS residents to better understand how the COVID-19 pandemic has impacted their residency education from the viewpoint of current trainees in the United States. This nationwide survey was sent to all OS residents in American Accreditation Council of Graduate Medical Education (ACGME) accredited programs.

Before the study, the authors of this study had hypothesized that most OS respondents would perceive the COVID-19 pandemic as having a negative impact on their residency education regarding both surgical case volume and didactic education. Additionally, they hypothesized that many survey respondents would regard their overall well-being as having been negatively impacted.

## METHODS

### Participants and Study Design

Since participation in this study was anonymous, voluntary and information was kept de-identified, this study was determined to be exempt by the authors’ Institutional Review Board. U.S. accredited OS residency programs were identified using the public database, Fellowship and Residency Interactive Database (FREIDA™), sponsored by the American Medical Association (AMA).^7^

In an attempt to exclude any non-trainees from participating in the anonymous survey, program coordinators for all 201 registered OS residency programs (representing approximately 3,640 active residents) received a cover letter email including an electronic link to the anonymous Qualtrics survey and were asked to forward this link to their residents.^8–10^

Responses to the survey were accepted between April 20, 2020 and May 3, 2020. The responses remained anonymous and were stored on a secure server affiliated with the authors’ institution. The authors’ analysis included responses from all residents in program year levels PGYI-V who were in an ACGME-accredited OS residency program in the academic year of July 1, 2019-June 30, 2020.

The single 15-item study survey had been developed and agreed upon by authors DH and AZ to study resident perceptions of presumed changes on their residency experiences. Questions were previously approved by all authors. The survey also contained basic demographic questions as well as questions designed to assess resident experiences during the COVID-19 pandemic using a Likert-type scale.

## RESULTS

A total sample of 309 OS residents completed the survey. Assuming all 3,640 OS residents had been provided the opportunity to participate, this yields an approximate 8.5% response (309/3640). 230 of 309 (74.92%) of respondents were male, 78/309 (25.08%) were female and one respondent declined to indicate their gender affiliation. In terms of preparation level, 63 (20.39%) respondents were program-year level 1 (PGY-1), 60 (19.42%) PGY-2, 78 (25.24%) PGY-3, 60 (19.42%) PGY-4 and 48 (15.53%) PGY-5.

A subgroup of 153 respondents (49.52%) considered their program to be in an epicenter of the COVID-19 pandemic. 262 respondents (84.79%) reported that their program had adopted a “surge plan,” in which some residents were assigned clinical duties while others remained at home. Most (283 (91.58%)) respondents reported their residency clinical duty hours had decreased during the pandemic, with 300 (97.09%) reporting a decrease in surgical caseload.

A smaller proportion of 13 respondents (4.21%) reported they expected they would have difficulty achieving ACGME case requirements due to the pandemic, with an additional 29 residents (9.39%) unsure whether they would. Approximately a quarter of responding residents (27.18%) reported that the pandemic would cause changes in their future rotation schedules, while an additional 77 residents (24.92%) were unsure if there would be changes in their future rotation schedules.

A subset of 47 respondents (15.21%) reported that they had already been deployed to non-orthopaedic services because of the pandemic, with an additional 43 respondents (13.92%) reporting that they had not yet been deployed to non-orthopaedic services. Most residents (n=285, 92.23%) either “Somewhat” or “Strongly Agreed” that their program had enacted specific clinical policies and changes with their safety and well-being in mind.

**Figure 1.**
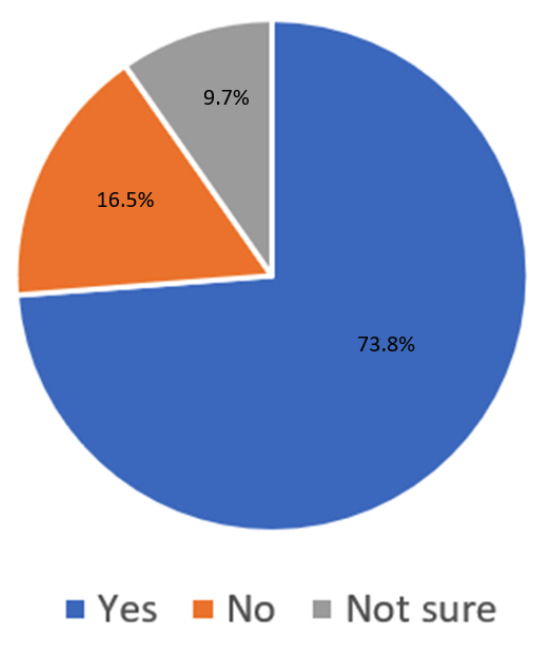
“Has your institution provided enough personal protective equipment (PPE) for hospital staff?” n=309 OS Residents

Notably, of the 47 respondents that had already been deployed to non-orthopaedic services, 40 (85.10%) agreed that their program had enacted changes with their well-being in mind. In comparison, of the 262 respondents who had not been deployed to non-orthopaedic services, 245 (93.51%) agreed that their program had enacted changes with their well-being in mind. A majority of 228 (73.8%) residents reported that their institution had provided enough personal protective equipment (PPE) for hospital staff (other proportions depicted in Figure 1).

**Figure 2.**
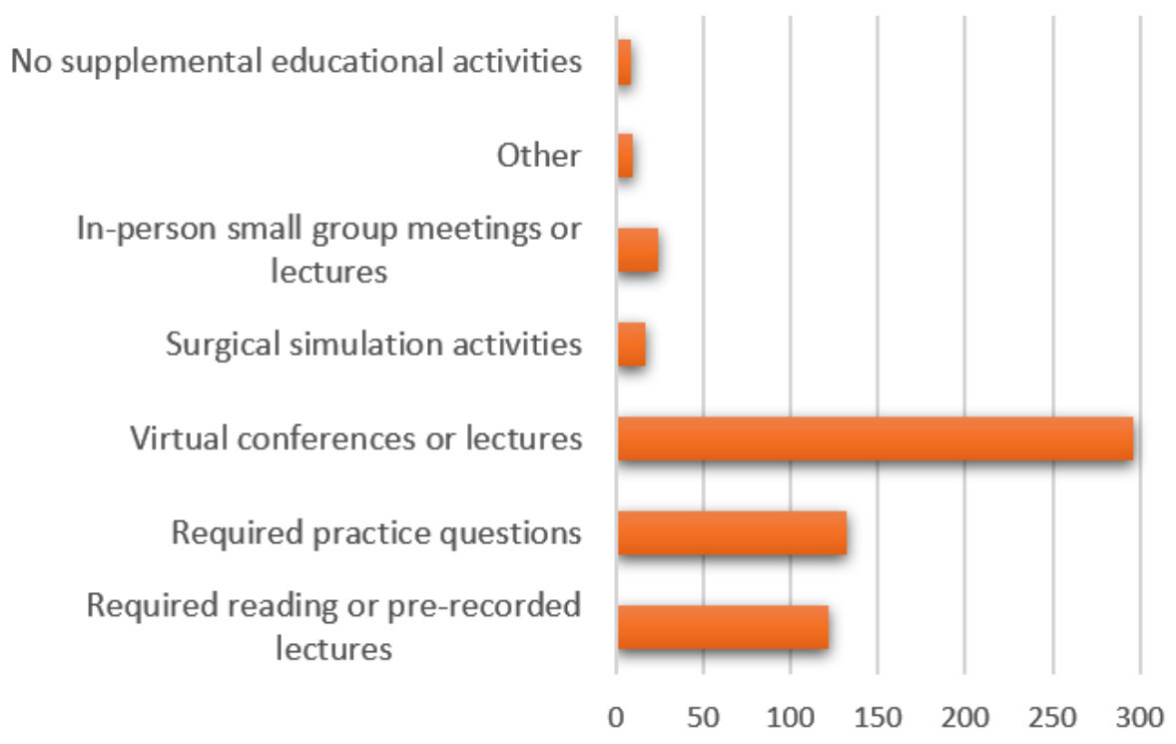
What has your program done to supplement your educational activities? n=309 OS Residents

Almost all residents (n=293, 94.82%) indicated that their institution had enacted curricular changes to supplement their GME educational activities due to the pandemic, the most common being virtual and video conferences (95.5%), required practice questions (42.7%), required reading or pre-recorded lectures (39.5%), in-person small group meetings or lectures (7.77%), and surgical simulation activities (5.50%). Only two residents (0.65%) volunteered that their institution had held in-person or virtual journal clubs. (Figure 2).

**Figure 3.**
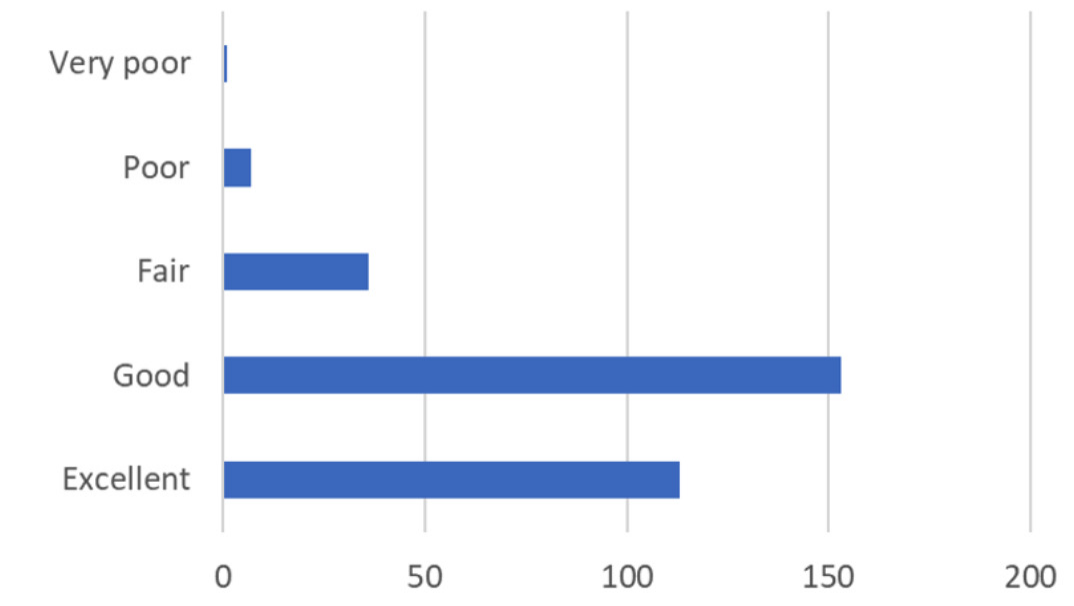
How would you grade your program’s adaptations to educational activities due to the COVID-19 pandemic? n=309 OS Residents

When asked to grade their program’s adaptations of GME educational activities due to the COVID-19 pandemic, 265 (85.76%) residents notably indicated either “Excellent” or “Good” (Figure 3).

Over half of residents reported that their personal and independent study hours had “Increased Greatly” (n =185, 59.87%) or “Slightly” (n=82, 26.54%) during the pandemic, with a small number indicating that these hours had “Decreased Slightly” (n=10, 3.24%) or “Decreased Greatly” (n=8, 2.59%). When asked how their overall resident education has been impacted by COVID-19, 28 (9.06%) said that it was “Much Better,” 56 (18.12%) said “Somewhat Better,” 74 (23.95%) said “About the Same,” 125 (40.45%) said “Somewhat Worse,” and 26 (8.41%) said “Much Worse.”

**Figure 4.**
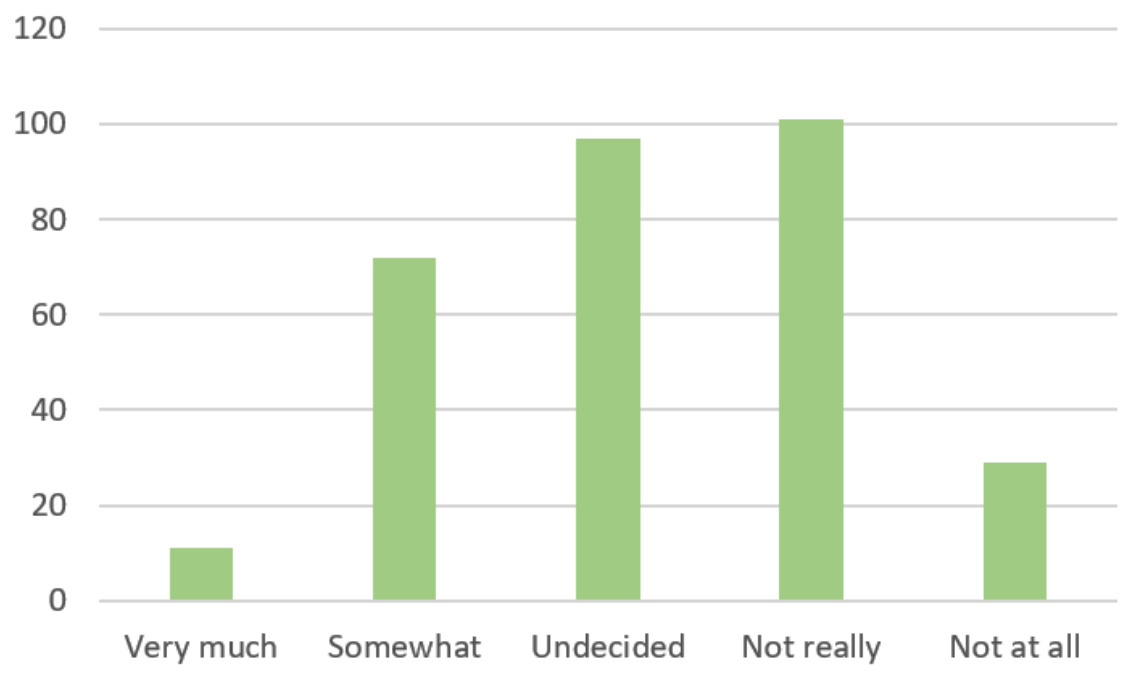
Have changes in your educational or clinical activities due to COVID-19 negatively impacted your overall competency in Orthopaedic surgery? n=309 OS Residents

A minority of 83 (26.86%) total residents reported that the changes that had been made in their educational or clinical activities had “Very Much” or “Somewhat” negatively impacted their overall perceived OS competencies (Figure 4).

**Figure 5.**
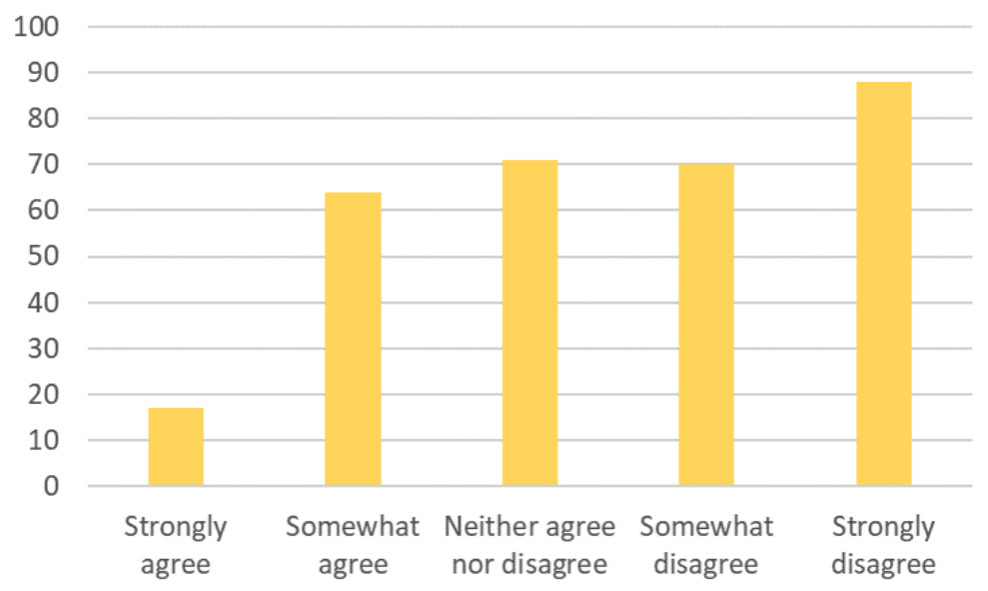
My well-being has been negatively impacted due to residency-related changes caused by the COVID-19 pandemic. n=309 OS Residents

Comparatively, of the 109 senior-level (i.e., PGY4 and PGY5) respondents, 25 (22.94%) reported that their perceived clinical competencies remained unchanged 81 residents (26.21%) “Strongly” or “Somewhat Strongly” agreed that their well-being had been negatively impacted by the pandemic (Figure 5).

## DISCUSSION

Based on these study results and the findings in other projects, the COVID-19 pandemic has created significant changes to the healthcare system in the United States and globally.^11,12^ Following the recommendations of the ACS and AAOS, elective surgeries were deferred at the onset of the pandemic and were under regional control by government officials.^3^ Many departments adopted surge plans in order to ensure that emergent care could continue to be provided.^13^

As evidenced in our study, over 84% of survey respondents had reported their residency program had adopted some form of a surge plan. Healthcare workers in all areas had also been presumably deployed to different services to help aid overflow care. To comply with governmental social distancing orders, educational and academic activities were either cancelled or substantially modified. These changes appear to have profoundly impacted nearly every aspect of the OS residency experience, including educational, clinical, and surgical activities.^14^

In response to social distancing recommendations and orders that prohibited normal academic and educational activities and conferences, several other authors have published recommendations for resident education that would allow for virtual or independent learning.^14–16^ Such recommendations included online video conferences, surgical simulation, and independent study activities. In our study, a resounding 94.82% of OS residents reported that their program had made changes to supplement their educational activities due to the COVID-19 pandemic, with almost all residents reporting virtual conferences as their main educational modality during the pandemic.

In our study, residents generally expressed favorable opinions of these changes, with over 85% of respondents evaluating their GME program’s adaptations to educational activities as “Good” or “Excellent.” The COVID-19 pandemic has accelerated the adoption and development of novel GME instructional methods of education for use during large national conferences, education, and networking.^17,18^

Despite the widespread adoption of virtual learning activities and increase in personal study habits, this pandemic has removed many residents from their usual hospital environments. Virtual learning, although beneficial, is an educational delivery method in stark contrast to hands-on learning typically associated with hospital and clinic settings. Such hands-on learning has been dramatically decreased due to several factors.^6,14^

Postponing elective surgical cases during the COVID-19 pandemic has led to a stark decrease in orthopaedic clinical care and case volume.^19^ This decrease in case volume has been compounded by stay-at-home orders that have substantially decreased emergency department visits by up to 42% compared to the year 2019, presumably including orthopaedic trauma volume.^19^

It is not surprising that about 27% of our resident respondents believed that pandemic-related changes in their educational and clinical activities had at least somewhat negatively impacted their perceived competencies in OS surgery. Despite their usefulness, virtual learning activities can be host to inherent issues, including challenges in assessing technical and non-technical skills.^15^

As seen in these results, decreases in case volume (noted in our survey to be ~97%) and clinical activities (~92%) can be a notable concern for many OS residents at risk of not attaining ACGME minimum surgical case requirements or meeting American Board of Orthopaedic Surgery (ABOS) educational hour requirements.^20^ Furthermore, case volume changes may cause residents to feel uncomfortable with the level of OS surgical training and exposure that they have received.

Our study results somewhat reflect these concerns, with 4.21% of respondents indicating that they would have challenges in achieving their ACGME case requirements. In 2020, the ABOS noted that they were willing to work with programs on a case-by-case basis for issues related to these minimum hour requirements.^20^ In addition, the determination of whether a resident/fellow can graduate as previously scheduled is still the responsibility of the program director.^18^ With pandemic changes, the determination of whether or not a resident has met the criteria for graduation can still be made although the curriculum as originally planned was not completed.^21^

The COVID-19 pandemic has already been shown to create substantial changes in the personal, economic, and societal circumstances of healthcare professionals.^22^ The impact of pandemic-related changes may be amplified for physician residents.^15,23–26^ It should obviously be concerning to program directors and faculty that over a quarter of respondents in this study reported that residency-related changes due to COVID-19 had a negative impact on their well-being.

### Study Limitations

First, although the survey was administered to every program in the United States, survey completion was voluntary, which limited the number of respondents and potentially created a selection bias in participants. In our recruitment strategy for this study, we also relied upon program coordinators at each program to forward our recruitment email to their residents. We presume many potential survey participants never received our survey. Additionally, this study only provides a snapshot of circumstances at the first wave of the COVID-19 pandemic.

## CONCLUSIONS

Based on these results, the COVID-19 pandemic has imposed substantial changes on the GME experiences of most OS residents. Although the majority of respondents in this study expressed favorable opinions of the educational changes that their GME programs have instituted, clinical duty hours and case volume were reported to have substantially decreased with a large number of residents viewing their resident education as having worsened and negatively impacting their well-being.

Future studies using more controlled analytics at the conclusion of the COVID-19 pandemic may provide longer-term data regarding changes in residency education and allow for program directors and board and accreditation bodies to evaluate their programmatic changes to facilitate improved resident performance, well-being, and remedy surgical caseload backlogs.^27^

### Conflict of Interest

None.
